# Albrecht von Graefe in the present, the past, and the future

**DOI:** 10.1007/s00417-020-04642-7

**Published:** 2020-03-07

**Authors:** Jens Martin Rohrbach

**Affiliations:** University Eye Hospital, Elfriede-Aulhorn-Straße 7, D-72076 Tübingen, Germany

**Keywords:** Albrecht von Graefe, Archive, Monuments, Scientific work, Influence

## Abstract

Albrecht von Graefe (1828–1870) is the founder of this archive (1854) and the founder of modern ophthalmology. In 2020, the anniversary of his death will be observed for the 150th time. The “German Ophthalmological Society” (DOG), also a Graefe foundation (1857), has therefore proclaimed a “Graefe year.” In Berlin, his hometown, several Graefe-monuments exist. Ophthalmology owes Albrecht von Graefe numerous first discoveries such as excavation of the optic disc in glaucoma (1855), iridectomy in glaucoma (1857), or central artery occlusion (1859). But his after-effects are not only based on his clinical and scientific merits but also on his extraordinary, fascinating personality, which can be characterized by his spirit of internationality, friendship, self-criticism, love of truth, and modesty. Graefe became a myth not only because of his early death but also because he had apart from great successes, to accept human misfortunes at the same time. Albrecht von Graefe can be regarded as the conscience of ophthalmology in Germany.

“What are all the goods of life, glory, worship, and what else appeals to men, against the happiness that friendship and love imposes on us. Without it, the soul burns and thirsts, and lacks the inner magic of sensation that makes us worth living; and to whom we can not think without the most painful melancholy, if we miss it. It is the real home of the heart, its loss unbearable exile. Every intimate relationship between people is a sanctuary because man is only through man” (Albrecht von Graefe to his friend Adolf-Schufft Waldau (1822–1895) from Isola bella in the Lago Maggiore/Italy, 1854, in the year of the foundation of this archive and the birth of his first (premarital) child [1].

This archive was founded in 1854 in Berlin by Albrecht von Graefe (May 22, 1828, Berlin–July 20, 1870, Berlin) [2–4] (Fig. 1) as an “Archive for Ophthalmology” (Fig. 2). After Graefe died of tuberculosis, co-editors and friends Ferdinand von Arlt (1812–1887) from Vienna and Frans Cornelius Donders (1818–1889) from Utrecht/Netherlands renamed the journal in 1871 to “Graefe’s Archive for Ophthalmology” (Fig. 3). A similar name is still used today, although the language is no longer German but English. “Graefe’s Archive” is the oldest worldwide ophthalmic journal to date.

**Fig. 1):**
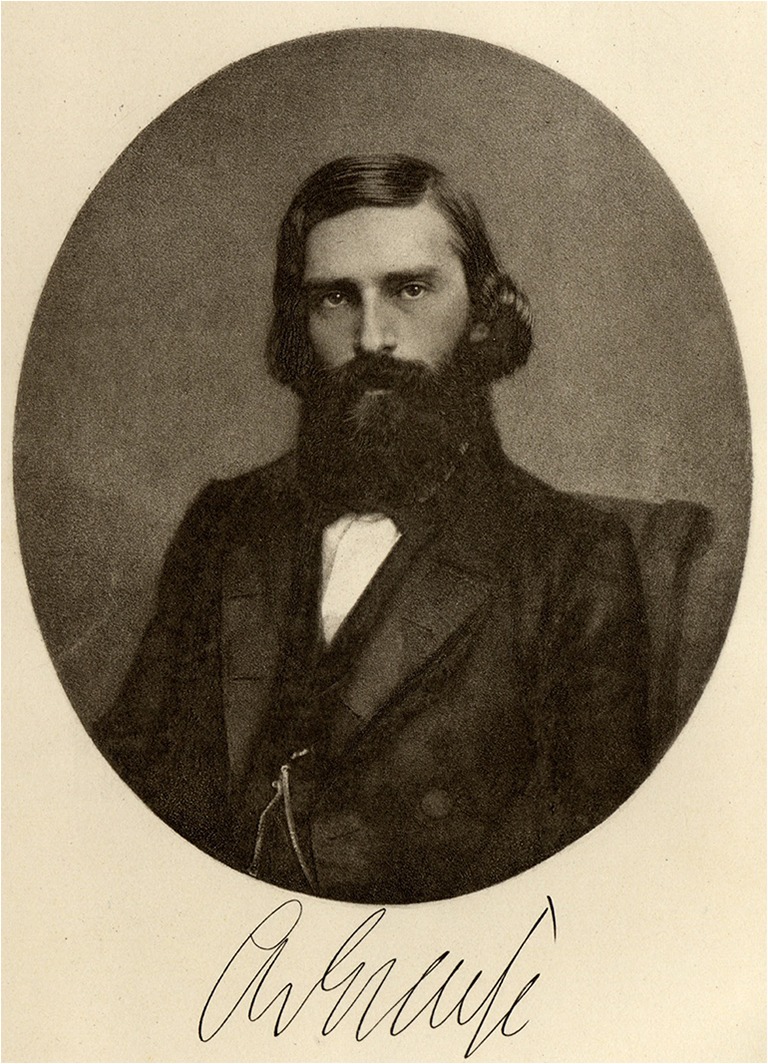
Albrecht von Graefe at the age of about 25 years [2]

**Fig. 2):**
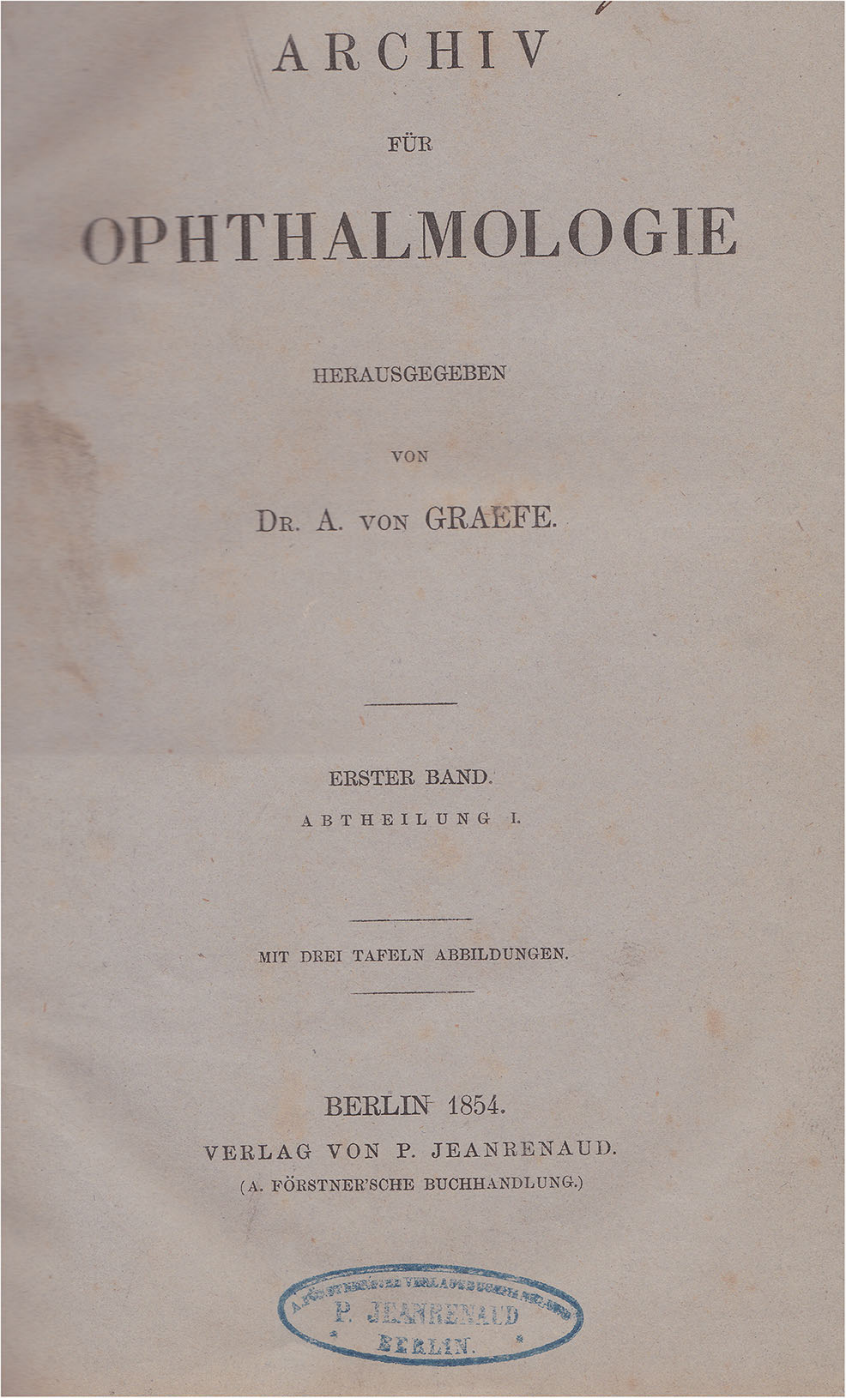
Volume 1 of the “Archive for Ophthalmology”, 1854, still without “Graefe”

**Fig. 3):**
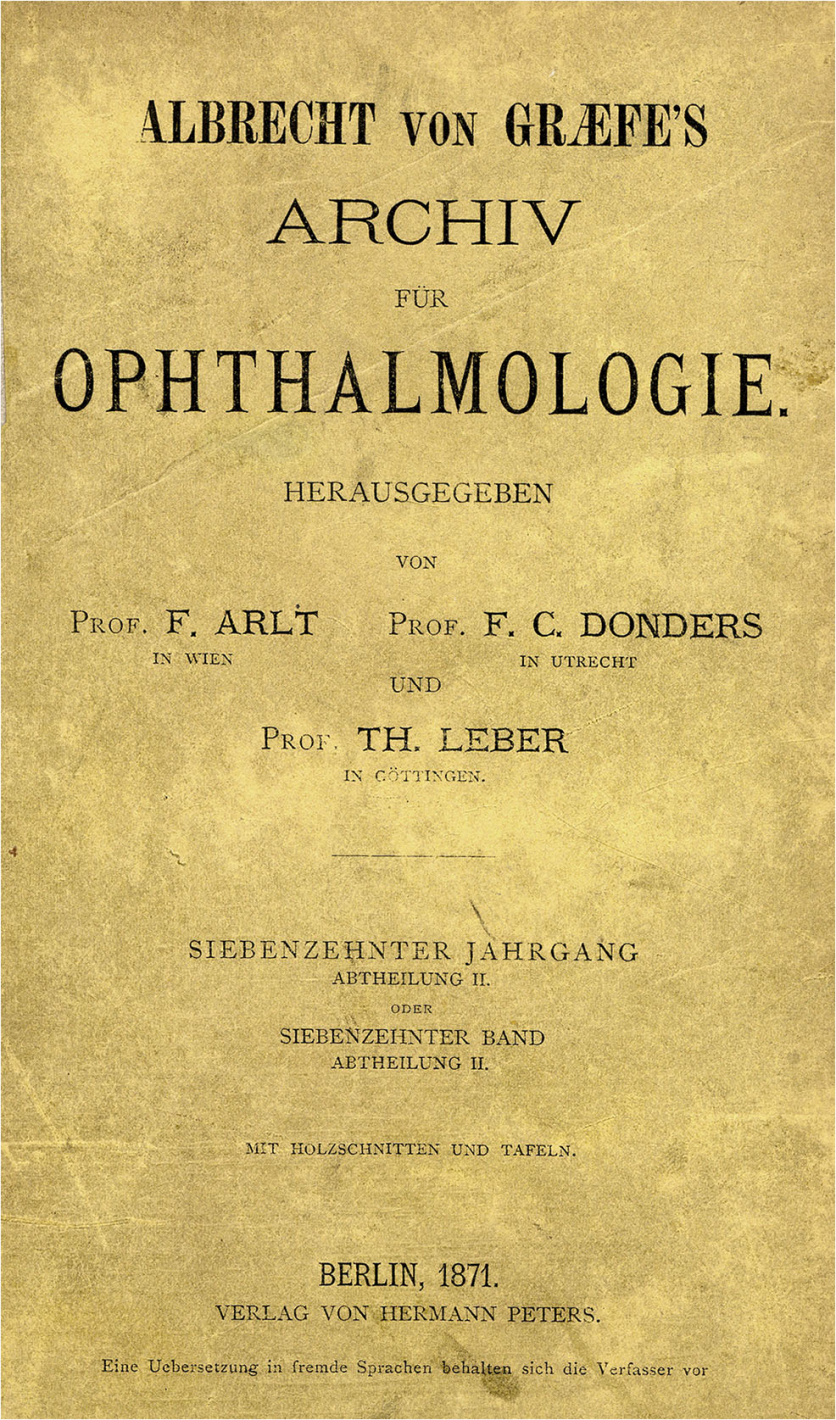
“Archive” of 1871, now, after his death, with the addition of “Graefes”

The year 2020 marks the 150th anniversary of Albrecht von Graefe’s death. The “German Ophthalmological Society” (DOG) was founded in 1857, 3 years after the “Archive,” also by Albrecht von Graefe, and is today the oldest ophthalmological society. It has proclaimed 2020 as a “Graefe year” in which various events are planned to remember the great ophthalmologist, scientist, Berliner, Prussian, European, and world citizen.

“Graefe’s Archive” and the DOG are today more vital than ever and remember Albrecht von Graefe daily. Every 2 years, the DOG awards a “Graefe-Preis” and every 10 years a “Graefe-Medal”. The latter is the highest award awarded by German ophthalmology. Of the 13 award winners so far—the first was Hermann von Helmholtz (1821–1894) in 1886 for the invention of the eye mirror—five came from abroad, as it corresponds to the international character of the DOG since its foundation. The “Berlin Medical Society”, whose first chairman was Graefe from 1860 to 1870, also honors deserving physicians with a Graefe medal.

In 1882, on his 54th birthday, the Graefe monument at the Charité was unveiled and inaugurated (Fig. 4). It was the first monument for a scientist in Berlin, donated by colleagues and friends from all over the world, including the Russian Tsar. The monument was badly damaged in the World War II and repaired by the German Democratic Republic (GDR). In 2005, it was restored among other things from donations of the DOG, so that it shines today in magnificent splendor and is always worth a visit for Berlin tourists.

**Fig. 4):**
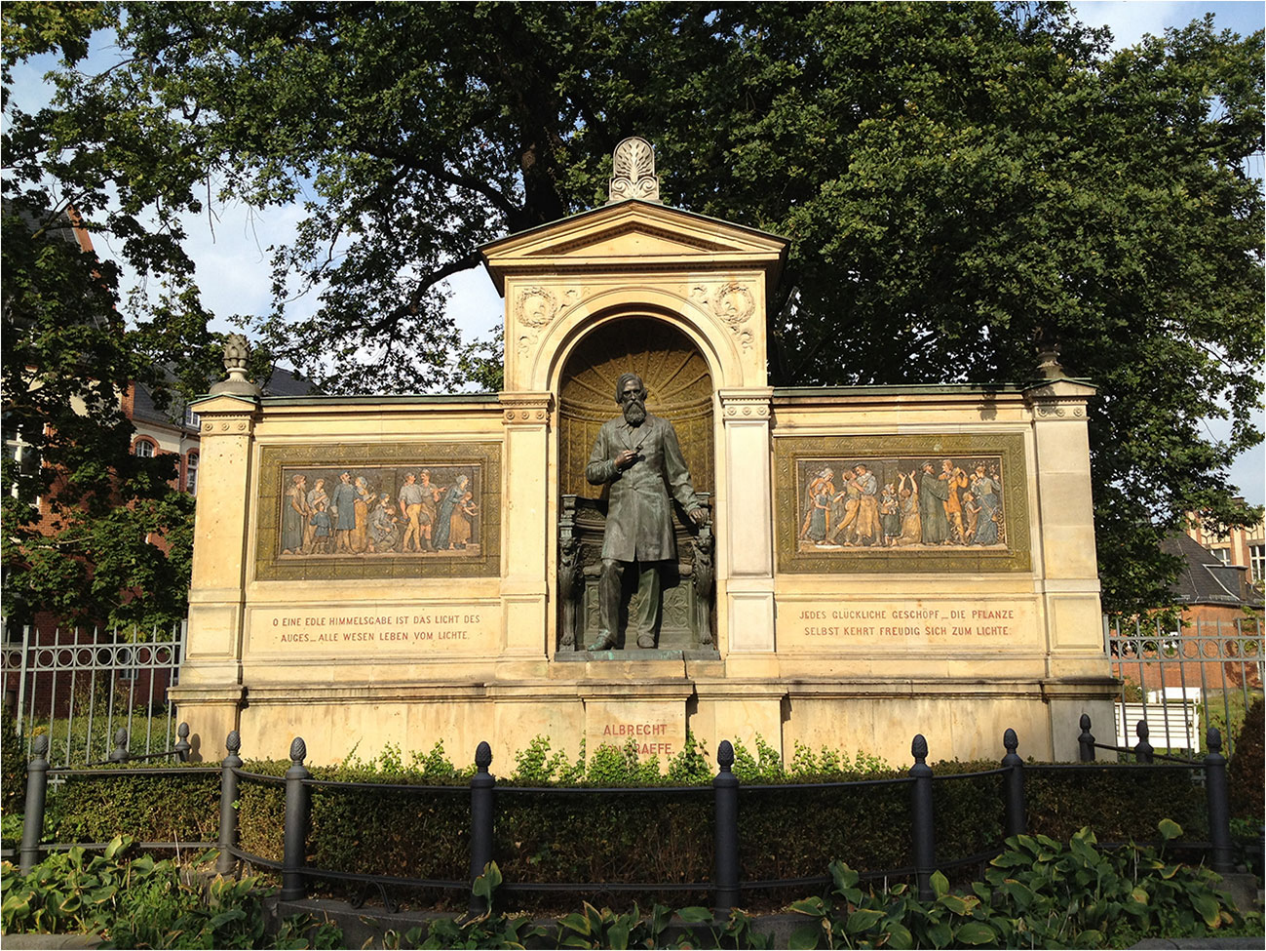
Graefe monument at the Charité. In his right hand Graefe holds an eye mirror of Helmholtz. The bronze statue and majolica reliefs depicting healing seekers and healing finders were created by the sculptor Rudolf Siemering (1835–1905). Unusual for the time was that the figure of Graefe was placed not on a podium, but in a niche. The verse “O a noble gift of heaven is the light of the eye” comes from Friedrich Schiller’s (1759–1805) “William Tell”

Although the monument stands on the edge of the world-famous Charité, founded in 1810, Albrecht von Graefe’s relationship with this institution was not straightforward. It was not until 1866 that Graefe received the chair of ophthalmology at the Charité, as this was until then occupied by Johann Christian Jüngken (1793–1875). In particular, in his letters to his pupil and friend Julius Jacobson (1828–1889) in Königsberg (now Kaliningrad, Russia), Graefe complained bitterly about the lack of equipment, the faculty, and the Prussian ministerial bureaucracy [5]. His invigorating “counterpoles” were his private clinic in today’s Berlin Reinhardtstraße, the DOG, and his—this—archive. With Jacobson, Graefe vehemently advocated for the separation of ophthalmology at all universities. However, he did not experience this realization. If today we are independent ophthalmologists and not appendices of surgery that is in no small way due to Albrecht von Graefe. His friend, the famous pathologist at the Charité Rudolf Virchow (1821–1902), was against the independence of ophthalmology.

In 1970, on the 100th anniversary of his death, a memorial was set up by the German ophthalmologists in the Tiergarten (“Animal garden”) in Berlin on the site of his birthplace “Finkenheerd,” which had been destroyed during the World War II (Fig. 5). The tomb of Albrecht von Graefe and his wife Anna (1842–1872) at the cemetery in Berlin-Kreuzberg is now being looked after by the city of Berlin as a grave of honor, which is next to the grave of his parents (Fig. 6). His father, Carl Ferdinand von Graefe (1787–1840), had been the first director of the department of surgery at the Charité. He worked intensively on ophthalmology and is considered the founder of the conjunctival etching with silver nitrate in gonoblenorrhea, which led to the introduction of the corresponding prophylaxis in 1880 by the Leipzig gynecologist Carl Sigmund Franz Credé (1819–1892).

**Fig. 5):**
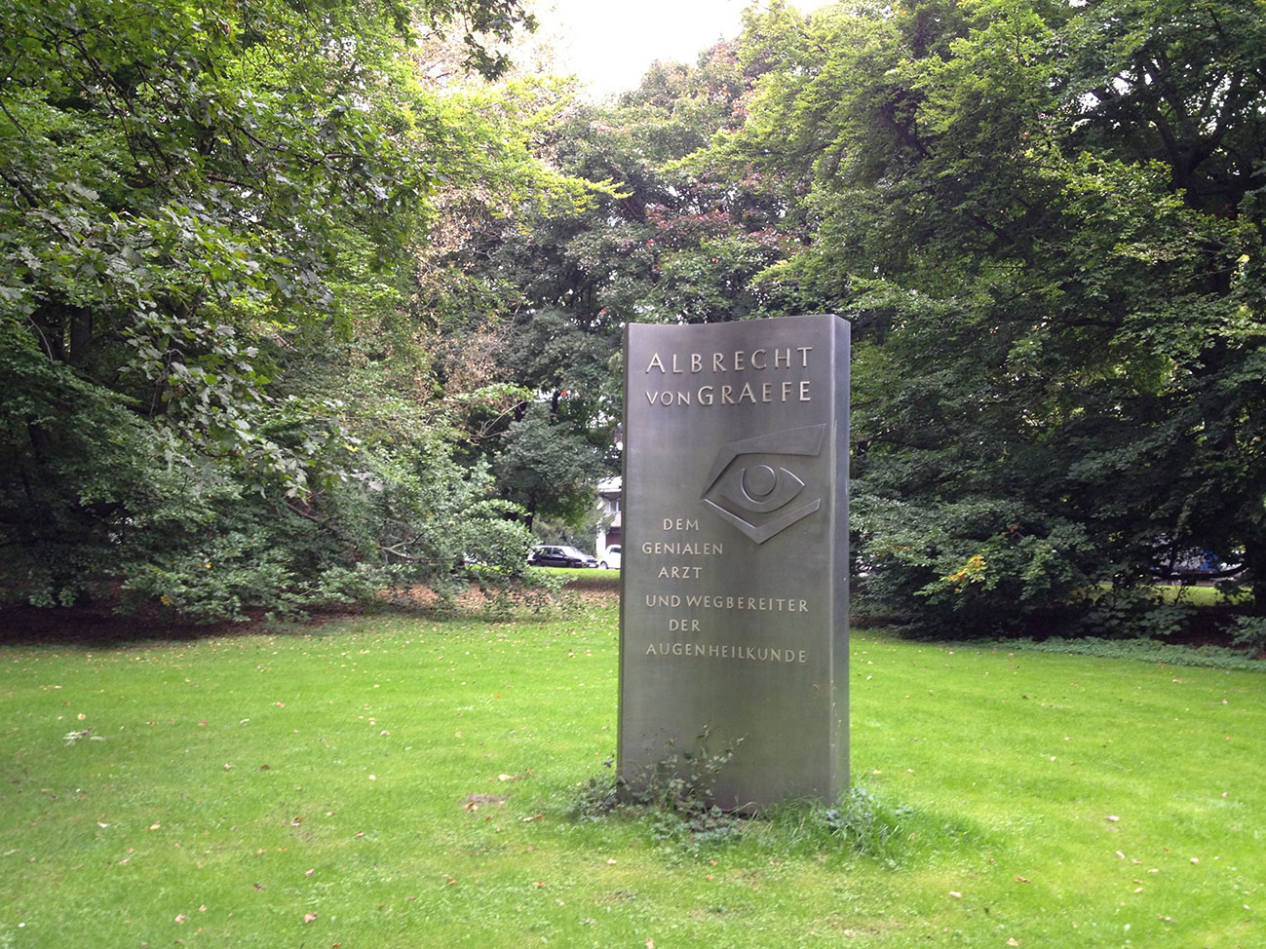
Commemorative stele in the Berlin Tiergarten from 1970 to commemorate the 100th anniversary of Albrecht von Graefe’s death with the inscription “To the brilliant physician and pioneer of ophthalmology”. The wave-like shape of the stele designed by Edzard Hobbing (1909–1974) symbolizes the light waves

**Fig. 6):**
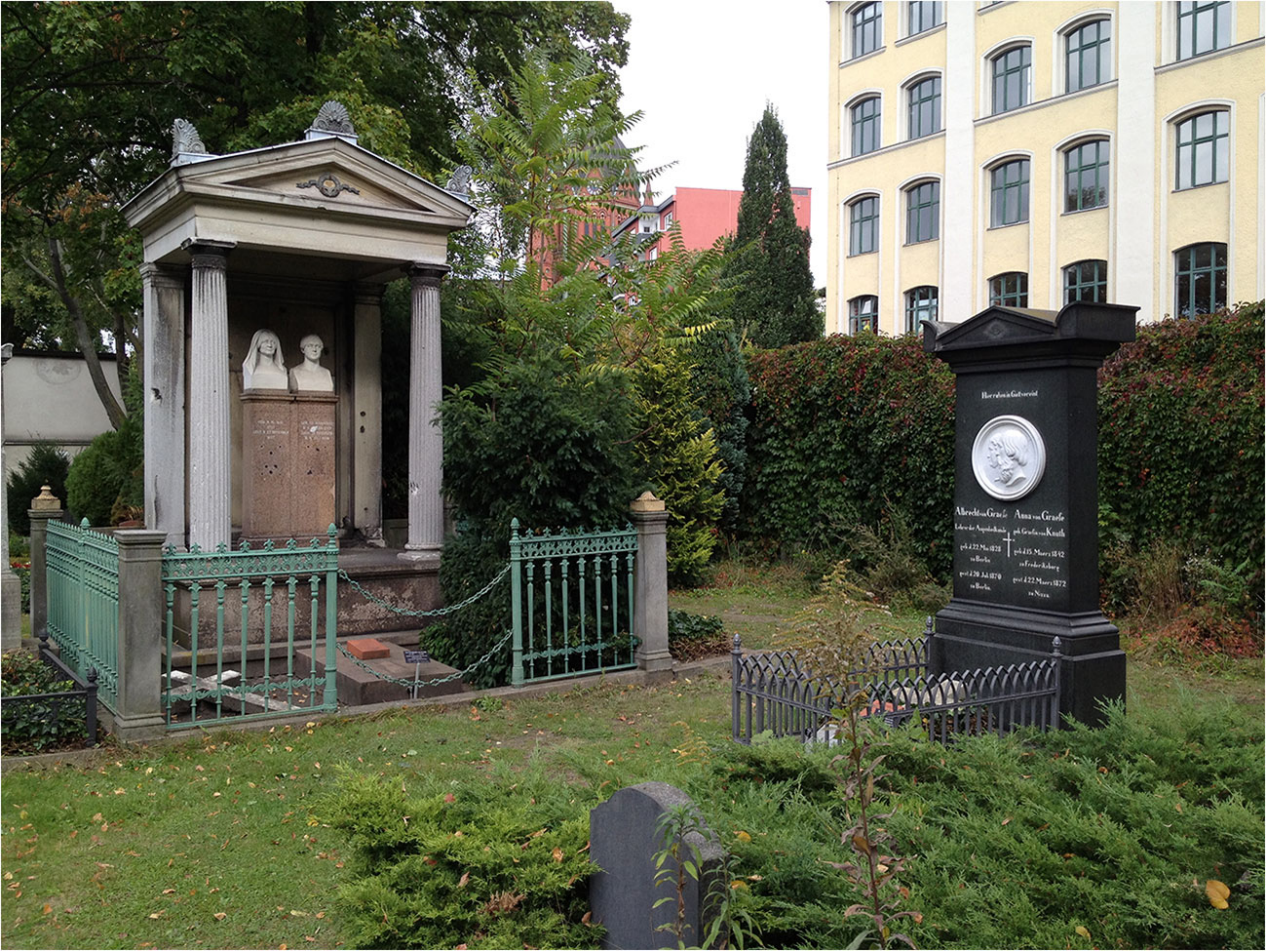
Tomb of Albrecht von Graefe and his wife (right picture edge) and his parents (left edge of picture) in the cemetery II of the Jerusalem and New Church parish in Berlin-Kreuzberg. On the back of the stele, there are the Bible texts “Love is strong as death” (Song of Songs) and “It is the light sweet and the eyes lovely to see the sun” (Preacher)

In 1820, Ferdinand von Graefe and Philipp von Walther (1782–1849) published the “Journal of Surgery and Ophthalmology” for some years (Fig. 7). His son Albrecht was given the publication of an ophthalmological journal, this archive.

**Fig. 7):**
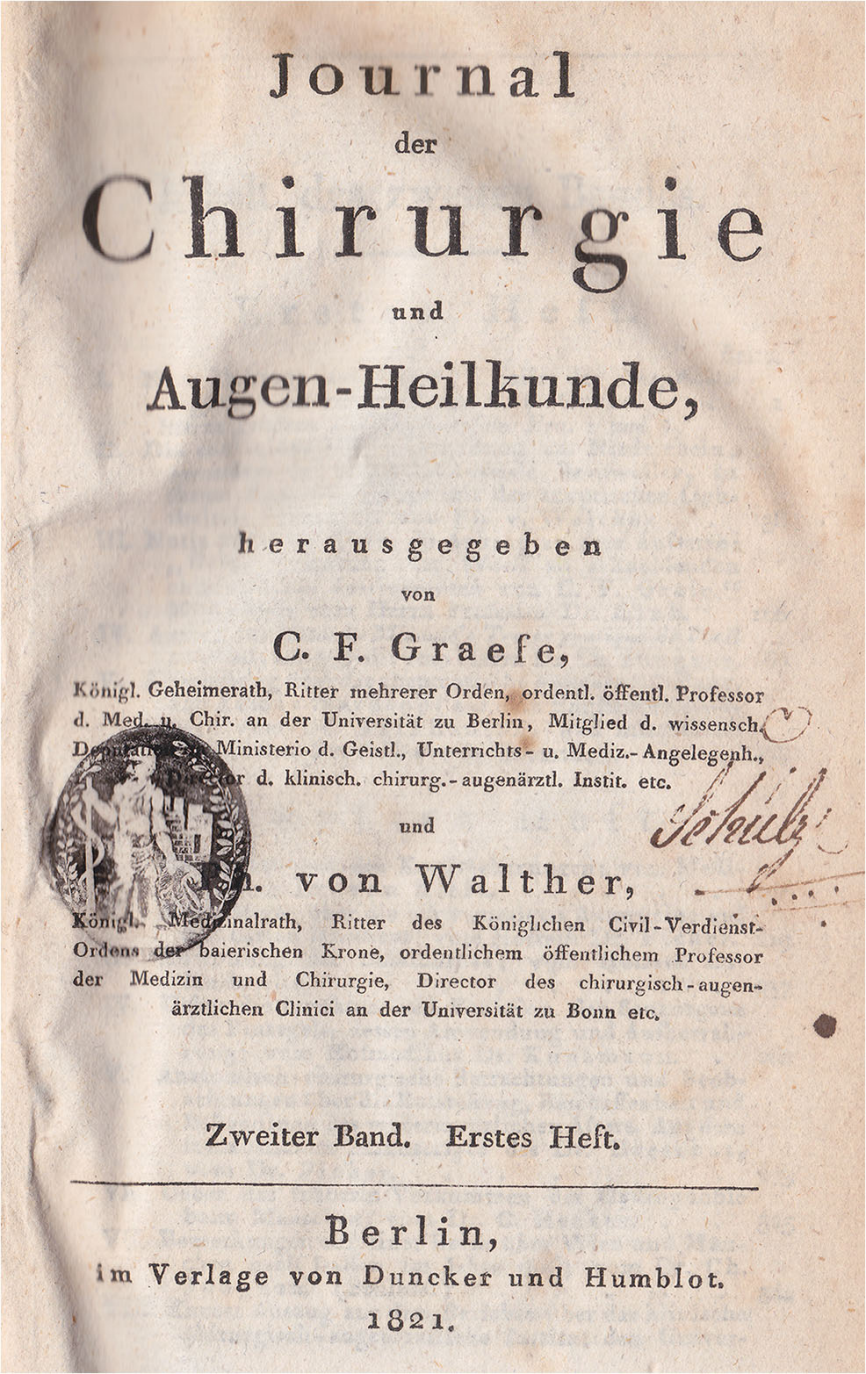
“Journal of Surgery and Eye Medicine”, 2nd volume of 1821. The father of Albrecht von Graefe, Carl Ferdinand, was responsible as publisher. If you like, this magazine, which only appeared for a few years, was the predecessor of today’s “Graefe’s Archive”

In Heiden, in the Appenzeller Land (Switzerland), where Graefe recovered very often in autumn, there is today a Graefe trail and a Graefe stone. In 1875, a street in Berlin-Kreuzberg was named after Albrecht von Graefe. It is there today. In 2015, the “Albrecht von Graefe-School” located in this street was officially named after the founder of modern ophthalmology. So even today, people who are not directly involved in ophthalmology are responsible for keeping the memory of Albrecht von Graefe alive.

Of course Albrecht von Graefe also lives on in the literature. Numerous biographies have been published, the first in 1877 [3], the last one so far in 2013 [4]. A new biography, based primarily on quotes from Graefe and his friends and colleagues, is due to be released in April/May 2020. In addition, letters of Graefe to various colleagues, such as to Adolf Schufft-Waldau [1], Julius Jacobson [5, 6], and Cornelius Donders [4] as well as collections of his lectures have been edited [7].

Graefe was already an “early media star”, although he probably would not have wanted that owing to his modesty. The illustrated magazines that emerged in the middle of the nineteenth century adopted him and his private eye clinic, founded in 1852 (Fig. 8) [8]. In addition, Graefe was famous not only during his lifetime, but posthumously for characters in novels, such as in “Behind blue glasses” by Friedrich Wilhelm Hackländer (1816–1877) from 1869 [9], or in Karl May’s (1842–1912) “Waldröschen” (“Wildflower”), which appeared as a serial novel from 1882 to 1884 [10]. These works are now largely forgotten. But if one reads them, one will inevitably be reminded of Albrecht von Graefe.

**Fig. 8):**
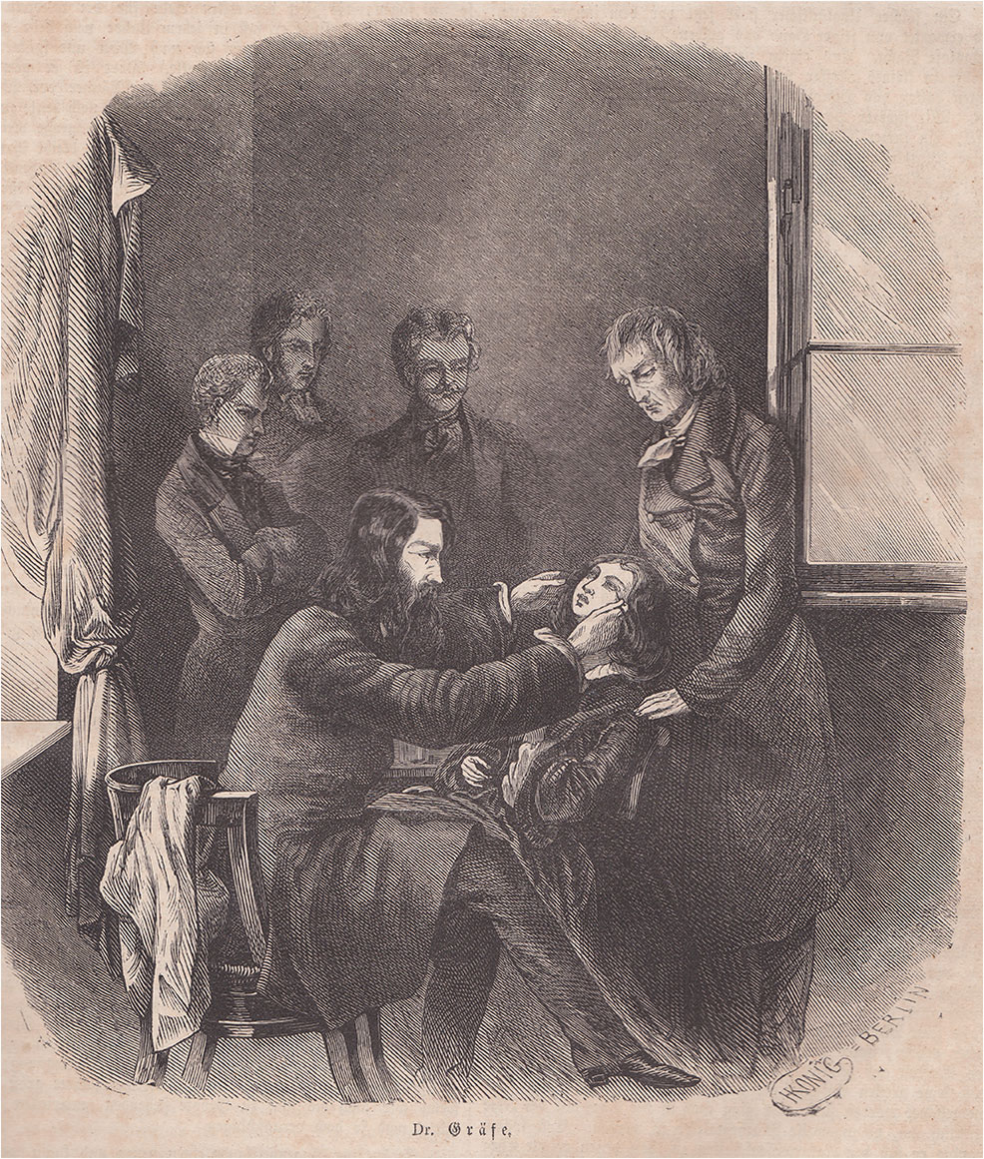
“Dr. Gräfe” in his clinic during an operation. The picture was published in 1857 in the then best-known illustrated tabloid “Die Gartenlaube” (“The Gazebo”) [8]. The text for the drawing was written by the physician, journalist and Graefe friend Max Ring (1817–1901). Other reports on Graefe with a drawing appeared in the illustrated magazines “Daheim” (“Home”) 1865, and “Über Land und Meer” (“Over Land and Sea”) 1869

Many scientific papers still testify to Graefe’s creative power. Between 1854 and 1869, he filled about 2500 pages of his archive in large part with his “little messages”, that is, case reports to which he attached special value. First descriptions, which all appeared in the “Graefe archive” are as follows:

- 1855 the excavation of the optic disc in glaucoma [11]
- 1857 the iridectomy for the treatment of glaucoma [12]
- 1859 the central artery occlusion [13]
- 1860 the Neuritis nervi optici in “brain diseases” [14]
- 1866 the swelling of the optic disc with increased intracranial pressure [15]
- 1868 the choroidal changes in tuberculosis together with his pupil Theodor Leber (1840–1917) [16]

Graefe dealt intensively throughout his life with the strabismus, especially the effect of the oblique eye muscles [17, 18], his habilitation in 1852. He owed many of his discoveries to Helmholtz’s ophthalmoscope of 1851. But even without this invention he would have become a great man of our field. He also dealt with the improvement of cataract surgery. Not least because of this, the American Society of Cataract and Refractive Surgery (ASCRS) included him into their “Hall of Fame” in 2000 [19]. Graefe addressed almost all facets of ophthalmology, with ophthalmopathology and general disease relationships being particularly important for him [20]. Although he considered himself a “specialist in ophthalmology”, he always saw ophthalmology as part of all medicine. A former, (no longer in use today) knife for cataract surgery with a narrow blade, a lid mark (retention of the upper eyelid in lowering the eye) in endocrine orbitopathy, and the ophthalmoplegia externa progressiva still bear his name today.

Let us examine the ideas and values Albrecht Von Graefe left us. First of all, there is the spirit of internationality [21], which certainly had something to do with the fact that his father came from Poland and his wife Anna from Denmark. Before he started his ophthalmological practice, he visited Prague, Vienna, Paris, and London to be there with the time-acknowledged magnates of ophthalmology. Graefe had attended the French school in Berlin and was Francophile throughout his life. He was fluent in English but more so in French. Vacation trips repeatedly took him to France, Italy, and Switzerland. He had numerous international friends beyond Arlt and Donders, just to mention two of the well-known ophthalmologists, Friedrich Horner (1831–1886) from Zurich and William Bowman (1816–1892) from London. Thanks to Graefe, the Heidelberg congresses of the DOG were visited by international colleagues right from the start. Graefe had many patients from abroad. Colleagues also came from other countries and continents to learn in his private clinic for a few weeks or months. The brilliant ophthalmo-historian Julius Hirschberg (1843–1925), one of his most important disciples, rightly described Graefe as the “teacher of the world circle” [2]. Julius Jacobson said:

“By all means of science, unfortunates should be helped far beyond the bounds of his sphere of influence, the eyewitnesses of his activity and successes should be his deputies, the fruits of his labor should not be lost to men after his death. This purpose gave his teaching something peculiar, from all the limitations of the person, school, nationality free, over petty, self-righteous bickering a higher value, which involuntarily informed his listeners, of whatever nationality they were. The ‘schools’, the ‘national ophthalmologies’ vanished” [6].

Graefe was always in search of the “scientific truth”. He was always questioning himself, often correcting himself. His lectures were described by his students as captivating. Although he came from a very wealthy family, he was personally undemanding. He treated the poor free of charge. His purpose was primarily to help other people. He was fully aware of the importance of sight for humans. So he said in a lecture in 1867:

“So much for the organ which exerts an influence for the nourishment of our mind, for the foundation of our world-view, and for the relation of men among themselves, about whose extent the person in undiminished possession can barely give full account. Speakers have praised it, poets have sung it; but the full value of it is sunk into the mute longings of those who once possessed and lost it” [22].

Albrecht von Graefe participated for the last time in 1868 in a conference in Heidelberg. His opening speech can be designated as his legacy:

“Three years have passed, my esteemed colleagues and friends, since the Ophthalmological Society met for the last time in this city of muses (Heidelberg). On one occasion it was the turmoil of the war (German-Austrian War of 1866) that held us back; gloomy times, which filled the hearts of the Germans with deep melancholy and awakened in all of us the dim consciousness of how much we nineteenth-century people still lag behind the real aims of cultural-historical development. The second time it was an event of a joyous nature that distracted our steps (3rd International Ophthalmology Congress in Paris, 1867). In the west, on the Seine (Paris), a splendid bloom of peace had arisen, resplendent in the colors of all lands, with the fruits of all the heavens, it astonished and compelled us to admire what we owe to the great advances of civilization.

Two years, the extraordinary ones, have gone away, and as the return to old dear habits belongs to the best and most natural pleasures of existence, it resounded when this year took its start, at ophthalmologists’ stoves with double joy, after the double interruption: ‘This year is to Heidelberg!’

Certainly different in our views of life, in our actions and activities, but also presumably in our scientific convictions - but at least in the pursuit of truth, in the culture of knowledge, in love for our field, we are together anew, to state the progress of science in order to draw from the rich source of communal work and experiences in order to broaden our own horizon, to the extent of which the most unconscious but more dangerous barrier of individuality presses, together with old friends to liven up the picture of past beautiful days, to bring fresh strength, to bring freer minds with them, into the often oppressive atmosphere of recurring misgivings, troubles, worries which, despite all success, surround the faithful service of Asclepius” [23].

These sentences contain everything that Graefe understood as a person: love of peace, cultural awareness, internationality, joy over civilizational and scientific progress, recognition of different opinions, science and truthfulness, enthusiasm for ophthalmology, friendship, conviviality and happiness, longtime connection with friends, finally, humane medical practice.

The “myth Graefe” is based not only on the fact that he died too early like other myths, but above all on the fact that despite his great clinical and scientific successes, the splendor of his family and a very special personality, he lived with drastic human blows. His parents died early, two of his five legitimate children died in their first year of life, and by 1861 Graefe knew that his tuberculosis was life-threatening. With death before his eyes he still tried to relieve human suffering.

Due to his timeless valuable convictions, Albrecht von Graefe can be regarded as the conscience of ophthalmology in Germany. He still is an essential part of present ophthalmology, of its history, and thus in a sense, of the ophthalmological future [24]. He is present materially as well as ideologically in many respects and will most likely remain so. In memorializing Graefe, this archive plays a major role.
